# Capacity Building of Health Professionals on Genetics and Genomics Practice: Evaluation of the Effectiveness of a Distance Learning Training Course for Italian Physicians

**DOI:** 10.3389/fgene.2021.626685

**Published:** 2021-03-15

**Authors:** Giovanna Elisa Calabrò, Alessia Tognetto, Alfonso Mazzaccara, Donatella Barbina, Pietro Carbone, Debora Guerrera, Alessandra Di Pucchio, Antonio Federici, Walter Ricciardi, Stefania Boccia

**Affiliations:** ^1^Section of Hygiene, University Department of Life Sciences and Public Health, Università Cattolica del Sacro Cuore, Rome, Italy; ^2^Servizio Formazione – Presidenza, Istituto Superiore di Sanità, Rome, Italy; ^3^Direzione Generale Prevenzione Sanitaria, Ministero della Salute, Rome, Italy; ^4^Department of Woman and Child Health and Public Health – Public Health Area, Fondazione Policlinico Universitario A. Gemelli IRCCS, Rome, Italy

**Keywords:** distance learning, capacity building, omics sciences, problem-based learning, genetics, medical education, genomics literacy

## Abstract

**Background:**

The rapid spread of personalized medicine requires professionals to manage the “omics revolution.” Therefore, the genetics/genomics literacy of healthcare professionals should be in line with the continuous advances in this field, in order to implement its potential implications for diagnosis, control and treatment of diseases. The present study investigates the effectiveness of a distance learning course on genetics and genomics targeted at medical doctors.

**Methods:**

In the context of a project funded by the Italian Ministry of Health, we developed a distance learning course, entitled Genetics and Genomics practice. The course focused on genetic/genomics testing, pharmacogenetics and oncogenomics and was developed according to andragogical training methods (Problem-based Learning and Case-based Learning). We used a pre-test vs. post-test study design to assess knowledge improvement on a set of 10 Multiple Choice Questions (MCQs). We analyzed the proportion of correct answers for each question pre and post-test and the mean score difference stratified by gender, age, professional status and medical discipline. Moreover, the test was submitted to the participants 8 months after the conclusion of the course (follow-up), in order to assess the retained knowledge.

**Results:**

The course was completed by 1,637 Italian physicians, most of which were primary care physicians (20.8%), public health professionals (11.5%) and specialist pediatricians (10.6%). The proportion of correct answers increased in the post-test for all the MCQs. The overall mean score significantly increased, from 59.46 in the pre-test to 71.42 in the post-test (*p* < 0.0001). The comparison in test performance between follow-up and pre-test demonstrated an overall knowledge improvement.

**Conclusion:**

Genomics literacy among healthcare professionals is essential to ensure optimal translation to healthcare delivery of research. The results of this course suggest that distance-learning training in genetic/genomics practice represents an effective method to improve physicians’ knowledge in the immediate and mid-term time scale. A preprint version of this paper is available at: https://www.researchsquare.com/article/rs-10083/v1.

## Introduction

The last two decades were characterized by a “genetic revolution” that has given rise to the “omics sciences era” as a consequence of the spread of high-throughput investigation techniques, capable of generating enormous amounts of data related to the different hierarchical levels of biological complexity (DNA, mRNA, proteins, metabolites, etc.) (Schneider and Orchard, 2011).

The rapid spread of this new knowledge requires healthcare professionals to manage the possible application of the omics sciences, ranging from medical research advances to use in screening, diagnosis and prognosis of different pathologies (Anderson and Schrijver, 2010; Muntoni and Cross, 2015).

While several European countries implemented dedicated health policies in this area (Boccia et al., 2015), few countries have integrated Public Health Genomics in their healthcare offer (Mazzucco et al., 2017), such as Italy where personalized medicine was included in the National Prevention Plans since 2010 (Conference of Regions and Autonomous Provinces, 2013; Boccia, 2014; Boccia et al., 2014).

More recently, the “Italian National Plan for innovation of the Health System based on omics sciences” identified the educational efforts toward professionals, citizens and decision makers as a cornerstone for a proper implementation of omics sciences in healthcare (Boccia et al., 2017). In the context of Continuous Medical Education (CME), the Italian Ministry of Health has supported a course entitled “Genetics and Genomics practice” (Calabrò et al., 2019). With the purpose of training medical professionals in the responsible use of omics technologies, a distance learning method was chosen. In recent years this method has increasingly become part of medical education programs and, besides allowing to reach a high number of learners, has shown to be effective in the context of CME (Vaona et al., 2018).

Our study presents the project aimed at developing a distance learning course in genetics and genomics targeted at medical professionals and at evaluating its effectiveness in terms of knowledge improvement of participants after the course, and 8 months after its closing.

## Materials and Methods

### Elaboration of the Scientific Contents of the Course

The course included audio-video lectures and interactive clinical cases and was structured according to the main models of andragogical training (Problem-based Learning and Case-based Learning). The Problem-based Learning (PBL) is a training methodology that stimulates the participants to “learn to learn” by solving real-world problems that reflect their work context (Schmidt et al., 2011; Mazzaccara et al., 2013). The Case-based Learning (CBL) is a teaching methodology used in medical education as an aid in connecting theory to practice (McLean, 2016).

The content of the course and the delivery model were identified according to two previous literature reviews: the first identified the core competencies in genetics/genomics for non-genetics healthcare professionals (Tognetto et al., 2019) and the second assessed the most effective educational interventions for health professionals in the “omics sciences” field (Pastorino et al., 2018). The course topics were validated by a panel of expert geneticists involved as teachers of the course.

The general and specific objectives and the content of the course, including 9 case studies, are reported in Table 1.

**TABLE 1:** Objectives and content of the distance learning course “Genetics and Genomics practice.”

**General objective**	**Training of medical professionals (in particular primary care physicians) in the responsible use of “omics” technologies.**
Specific objectives	Identify the basic concepts of human genetics Describe the main genetic/genomic tests currently available and their application Describe the main applications of pharmacogenetic tests Describe the main applications of genetic/genomic tests in oncology Consciously manage clinical information, family history and genetic test results for optimal patient management (including possible specialist referral).
**Topics**	**Case studies**
Public health genomics	
Genetic tests in the clinical practice	Pulmonary disease, sinusitis, digital hippocratism (Atypical Cystic Fibrosis) Unilateral maculopathy and predictive tests (example of predictive tests aimed directly at consumers) Monitoring of pregnancy with “super-villocentesis” or “super-amniocentesis”
Pharmacogenetics	Hypersensitivity to warfarin Patient with insufficient response to antiplatelet therapy Abacavir hypersensitivity syndrome
Oncology genomics	Hereditary breast cancer Family history of multiple cancers Hereditary colon cancer
Integration of genetic tests into cancer screening programs	

*GPs, General Practitioners; FPs, Family Pediatricians.*

### Course Characteristic, Learning Methodology, and Participants

The distance course “Genetics and Genomics practice” was accessible free of charge on the Italian National Institute of Health e-learning platform (EDUISS)^1^. The Learning Management System (LMS) used was Totara Learn, that offered the technical resources to reproduce the selected methodological approaches (PBL and CBL).

The course was delivered from February 27th, 2017 to February 1st, 2018. The course, open to all physicians potentially involved in the prescription and/or interpretation of genetic tests, was primarily targeted at General Practitioners (GPs) and Family Pediatricians (FPs). The maximum number of subscribers was 3,500. Successful completion of the course included the release of 30 CME credits. Participants were expected to spend 30 h to complete the course and could access the course at any time.

According to the Italian regulation, ethics approval was not required for this study: by registering for the course on the online platform, the participants gave the consent to the use of their anonymous data.

The course was structured in four sections:

1. Introductive section: introduction to the course explaining its relevance, general aims and structure; general objectives of the course; guide for participants containing all the instructions to attend the course; preliminary self-assessment test to set the initial knowledge (pre-test) consisting of 10 Multiple Choice Questions (MCQs). No minimum score was required to complete the test.
2. PBL section (1 entire PBL cycle—7 steps): problem presentation and analysis, specific learning objectives identification, bibliographical references and list of useful web sites to be consulted, reading materials to deepen the topics of the course, audio-video tutorials by experts and the solution of the problem.
3. Case Studies section: exercises on 9 different clinical cases (Table 1). Each exercise consisted of an initial part with a dialogue between patient and doctor, where the case was examined and in-depth studies proposed, a central part with clinical information and a third part with another dialogue, where the doctor made the diagnosis based on the data collected. Eventually the participant had to pass a 4–5 question test to complete the exercise.
4. Conclusive section: post-test (same 10 MCQs set of the pre-test), final certification test, satisfaction questionnaire. Passing the final certification test, consisting of 90 MCQs (0 points for wrong answer—1 point for correct answer) was mandatory to complete the course and get the CME credits. Each learning objective was tested in a set of MCQs. The final certification test was passed with a score of at least 75% correct answers. Three passing attempts were allowed.

Some Authors pointed out that MCQs couldn’t be fully appropriate to assess the competences acquired through PBL approach, as it should be based upon performance and not only upon giving correct answers (Azer, 2003). Nevertheless, MCQs tests can be considered suitable for self-assessment especially when required to assess a large amount of knowledge, as in the case in study (Tabish, 2008). Using MCQs tests was also chosen since the assessment was directed to the levels of “understanding” and “remembering” (Anderson and Krathwohl, 2001). Answering all the questions was mandatory to complete every MCQs test and blank answers were automatically considered as wrong answers, assigning them 0 points. Therefore, all participants who accessed the tests answered each question.

The course methodology integrated the PBL (Schmidt et al., 2011; Mazzaccara et al., 2013) and the CBL (McLean, 2016), in order to satisfy the strong clinical orientation of the course. Over the years PBL has been adapted to the e-learning context and different learning models have been developed, depending on the level of interaction between participants and facilitator (Mazzaccara et al., 2013; Calabrò et al., 2019). In courses with high turnout, the participants follow the steps of the PBL by their own, even if small groups or facilitator is not provided. In this course, the entire PBL cycle was set up using platform tools such as feedback, web pages, quizzes. The first steps of the PBL cycle, consisting of problem analysis and learning objectives identification, were provided through an interactive tool that allowed to track the results provided by participants.

The case studies were realized through interactive exercises, consisting of clinical case audio-video presentations, animated slides, clinical notes and final questions on the case focal points.

### Data Collection

When registering for the course in the e-learning platform, the following demographic and professional information about the participants were collected: gender, age, region of residence; CME discipline; professional status (National Health Service—NHS employee, freelancer, private contractor with NHS, unemployed). A preliminary question on the previous learning experience on genetics was proposed only at the beginning of the course. Afterward, a test of 10 MCQs was performed in order to gain insight on genetic knowledge at course registration (pre-test, T0). The same set of 10 MCQs was administered after the course (post-test, T1) before the CME certification test and was sent by e-mail to be repeated also 8 months after the closure of the course (follow-up, T2) to those who had completed the course.

The pre/post-test consisted of 10 MCQs related to the different modules of the course. We included very specific items to test different knowledge components. Two questions for each specific learning objectives (Table 1) were included. For those completing the test at T2, an additional question on the acquired competence to meet the patients requests on genetic tests was administered. At the very end of the course, participants who successfully completed the final certification test (consisting of 90 MCQs) were also required to fill in a satisfaction questionnaire (SQ), consisting of 18 closed questions about the perceived quality of the learning methodology, the educational contents and the e-learning platform functioning.

### Statistical Analysis

We performed a descriptive analysis for demographic and professional information. The results of each question, for the pre-test, post-test and for the follow-up test, were reported as percentages of correct answers. The pre-test and post-test results were compared through the McNemar test. We calculated a score for the 10 MCQs by assigning 10 points for each correct answer. The average scores of the pre-test and post-test were compared by *t*-test for paired data for the eligible participants, being data normally distributed. Data were stratified by gender, age categories, region of residence (North, Center, South of Italy, and Islands), medical discipline and professional status. The discipline of the participants was analyzed reporting individually those disciplines with more than 3% participants of the total, while those for which a lower percentage was recorded were grouped as “other specializations.” The results of the follow-up test were analyzed by presenting the number and proportion of those who gave the correct answer to the test questions. The pre-test (T0) and follow-up results (T2) were compared through the McNemar test.

Statistical analysis was performed using the Stata software (StataCorp. 2013. Statistical Software: Release 13. College Station, TX: StataCorp. LP).

## Results

### Course Participants

**FIGURE 1:**
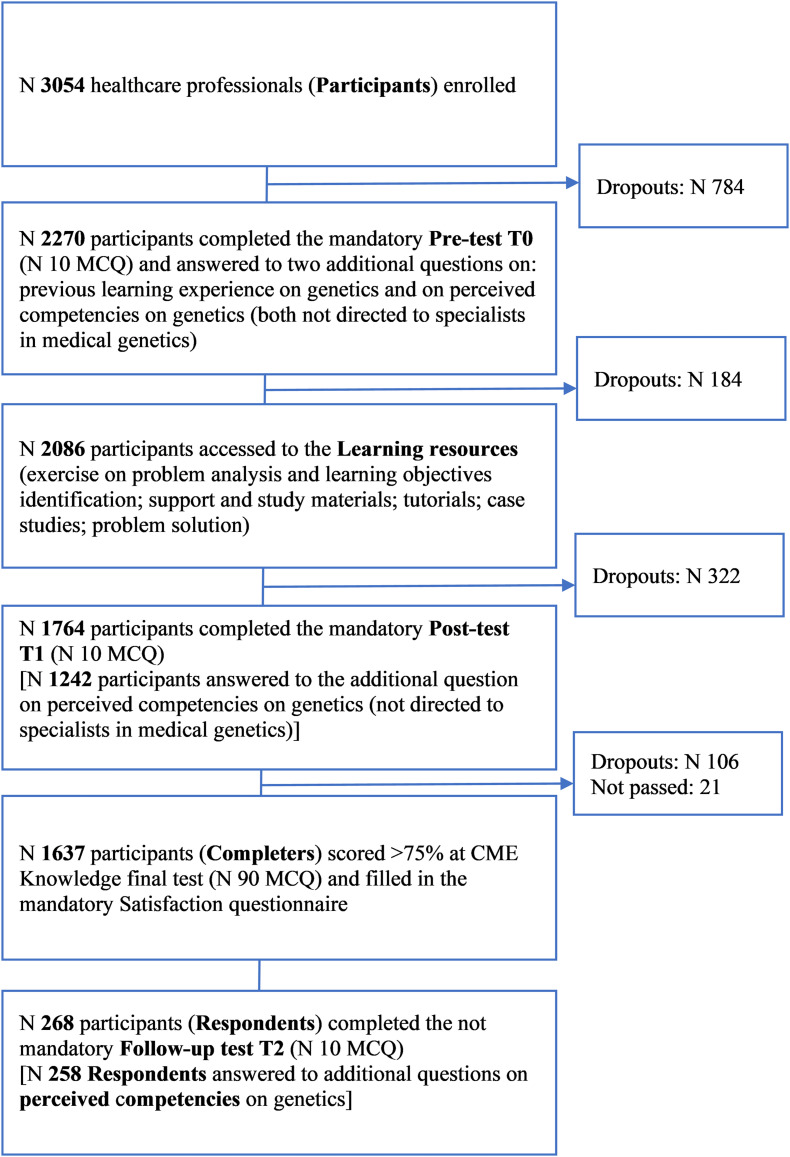
Flowchart of cohort selection of participants. CME, Continuous Medical Education; MCQ, Multiple Choice Questions.

The participants who completed the course (Completers) were 1,637 out of 3,054 physicians enrolled (Figure 1).

Table 2 describes the characteristics of the Completers and of the 268 participants who filled in the follow-up test (Respondents).

**TABLE 2:** Characteristics of the participants who completed the course (*N* = 1637) and of the Respondents at follow-up (*N* = 268).

	**Completers *N* = 1,637**	**Respondents at follow-up *N* = 268**
**Gender**	**Number (%)**	**Number (%)**
Male	790 (48.3)	145 (54.1)
Female	847 (51.7)	123 (45.9)
**Age (years)**		
<36	111 (6.7)	11 (4.1)
36–50	458 (28.0)	63 (23.5)
51–65	954 (58.3)	171 (63.8)
>65	114 (7.0)	23 (8.6)
**Italian region area**		
North	594 (36.3)	101 (37.7)
Center	413 (25.2)	67 (25)
South	630 (38.5)	100 (37.3)
**Medical specialty**		
Primary care	340 (20.8)	63 (23.5)
*GPs*	226 (13.8)	35 (13.0)
*FPs*	93 (5.7)	23 (8.6)
*Continuity of care physicians*	21 (1.3)	5 (1.9)
Hygiene and public health	190 (11.6)	40 (14.9)
Pediatrics	174 (10.6)	29 (10.8)
Psychiatry	118 (7.2)	19 (7.1)
Sports medicine	68 (4.2)	12 (4.5)
Genetics	60 (3.7)	1 (0.4)
Occupational health medicine	54 (3.3)	12 (4.5)
Gynecology and obstetrics	53 (3.2)	3 (1.12)
Other specialties	580 (35.4)	95 (35.5)
**Professional status**		
Private health facilities/NHS Employees	913 (55.8)	148 (55.2)
Freelancers	337 (20.6)	49 (18.3)
Private contractors with NHS	359 (21.9)	64 (23.9)
Without occupation	28 (1.7)	7 (2.6)

Among the Completers, 790 (48.3%) were male and 847 (51.7%) females. The median age was 56 years and the most represented age group was 51–65 years (58.3%). Most of the participants were from the South of Italy (including Islands) (38.5%), followed by North (36.3%) and Center (25.2%). The most represented disciplines were those referring to “primary care” (GPs, FPs, and Continuity of care Physicians), accounting for the 20.8% of participants, followed by “hygiene and public health” (11%), specialist pediatricians (10.6%), “psychiatry and psychotherapy” (7.2%), “sports medicine” (4.2%), “genetics and laboratory genetics” (3.7%), “occupational medicine” (3.3%), and “gynecology and obstetrics” (3.2%). Regarding the professional status, most of the participants were private health facilities Employees or NHS Employees (55.8%).

The Respondents at follow-up were 268 out of 1,637 (16.4%). Among them, 145 (54.1%) were males and 123 (45.9%) females. The mean age was 55 years and the most represented age group was 51–65 years (63.8%). Most of the participants came from the North of Italy (37.7%), followed by South (37.3%) and Center (25%). The most represented disciplines were “primary care” (23.5%), “hygiene and public health” (14.9%) and specialist pediatricians (10.8%). As for the professional status, most of the participants were private health facilities/NHS Employees (55.2%).

### Effectiveness of the Course

The preliminary question on a previous attendance to similar training courses indicates that 79.4% of participants had not attended other courses on the same topic before. The results of the pre-test vs. post-test comparison are reported in Table 3. A significant improvement was recorded in 100% of the questions.

**TABLE 3:** Knowledge level of the Completers before and after the course compared through the McNemar test (*N* = 1,637).

**N°**	**Question**	**Correct answers before the course (T0) N (%)**	**Correct answers after the course (T1) N (%)**	***p*-value**
1	Which of the following groups of diseases is characterized by the interaction between genes and the environment?	917 (56.0)	1,116 (68.2)	<0.0001
2	What is the name of the study of DNA polymorphisms in order to predict the safety and efficacy of drugs?	1,319 (80.6)	1,497 (91.5)	<0.0001
3	Is the evaluation of the hereditary-family risk of a tumor carried out as part of the oncological screening pathways?	515 (31.5)	866 (52.9)	<0.0001
4	What is a predictive test?	1,261 (77.0)	1,332 (81.4)	0.0026
5	What are the main models of Mendelian heritage?	1,446 (88.3)	1,522 (93.0)	<0.0001
6	What do pharmacogenetic tests predict?	1,263 (77.2)	1,479 (90.4)	<0.0001
7	What coverage does participation in cancer screening programs for breast and colorectal cancer reach in our country?	336 (20.5)	619 (37.8)	<0.0001
8	In hereditary forms of cancer, what is the transmission of the gene involved?	705 (43.1)	879 (53.7)	<0.0001
9	What types of analysis are performed with Next Generation Sequencing (NGS) techniques?	678 (41.4)	911 (55.7)	<0.0001
10	What is the role of diagnostic genetic tests in the field of hereditary tumors?	1,294 (79.1)	1,471 (89.9)	<0.0001

Table 4 presents the results of the comparison between average pre-test (T0) and post-test (T1) scores according to the participants’ characteristics. The average overall pre and post-test scores were 59.5 and 71.4, respectively, with a mean increase of 11.9 (*p* < 0.0001). In stratified analysis, a significant improvement in the average scores was recorded for all the categories considered. The stratified analysis by age shows that, with increasing age, the pre-test score was lower, along with a progressive increase in the difference between average pre-test and post-test scores. The stratification by region of residence demonstrates a North-South decreasing gradient both in the pre-test score and in the score increase between post-test and pre-test. The stratified analysis by medical discipline shows the greatest increase in knowledge for “sports medicine” physicians (score increase 15.3) and for “primary care” physicians (14.3). These classes had the lowest pre-test scores (53.1 “sports medicine physicians” and 57.0 “primary care” physicians). Within the “primary care” class, GPs had the lowest pre-test score (56.3) and achieved the greatest increase (score increase: 15.3) (data not shown). With regard to the professional status, private contractors with the NHS had the highest increase in scores (14.4) and the lowest pre-test score (57.9).

**TABLE 4:** Completers’ pre-test vs. post-test average scores according to several participants’ characteristics, compared by t test for paired data.

	**Pre-test (T0) mean score**	**Post-test (T1) mean score**	**Difference**	**p-value**
**Overall (*N* = 1,637)**	**59.46**	**71.42**	**11.96**	**<0.0001**
**Gender**				
Male (*N* = 790)	60.28	72.77	12.49	<0.0001
Female (*N* = 847)	58.70	70.17	11.46	<0.0001
**Age**				
<56 years (*N* = 814)	61.81	72.33	10.53	<0.0001
≥56 years (*N* = 823)	57.14	70.52	13.38	<0.0001
**Region**				
North (*N* = 594)	60.24	73.38	13.15	<0.0001
Center (*N* = 413)	59.54	71.94	12.40	<0.0001
South (*N* = 630)	58.68	69.24	10.56	<0.0001
**Medical specialty**				
Primary care (*N* = 340)	56.97	71.29	14.32	<0.0001
Hygiene and Public Health (*N* = 190)	60.21	70.63	10.42	<0.0001
Pediatrics (*N* = 174)	62.59	73.68	11.09	<0.0001
Psychiatry (*N* = 118)	57.97	70.68	12.71	<0.0001
Sports Medicine (*N* = 68)	53.09	68.38	15.29	<0.0001
Genetics (*N* = 60)	72.83	83.5	10.67	<0.0001
Occupational Health Medicine (*N* = 54)	58.89	68.33	9.44	0.0005
Gynecology and obstetrics (*N* = 53)	63.40	72.26	8.87	0.0017
Other specialties (*N* = 580)	59.10	70.55	11.45	<0.0001
**Professional status**				
Private health facilities/NHS Employees (*N* = 913)	59.55	71.04	11.49	<0.0001
Freelancers (*N* = 337)	60.68	71.39	10.71	<0.0001
Private contractors with the NHS (*N* = 359)	57.86	72.23	14.37	<0.0001
Without occupation (*N* = 28)	62.5	73.93	11.43	0.0039

Tables 5, 6 report the results of the Respondents at follow-up (T2) test.

**TABLE 5:** Knowledge level of the Respondents before (T0), after (T1) the course and at follow-up (T2) and comparison between T2 and T0 (*N* = 268), compared through the McNemar test.

**N°**	**Question**	**Respondents who gave correct answers at T0 N (%)**	**Respondents who gave correct answers at T1 N (%)**	**Respondents who gave correct answers at T2 N (%)**	**T2 vs. T0 *p*-value**
1	Which of the following groups of diseases is characterized by the interaction between genes and the environment?	150 (56.0)	190 (70.9)	180 (67.2)	0.0030
2	What is the name of the study of DNA polymorphisms in order to predict the safety and efficacy of drugs?	210 (78.4)	249 (92.9)	231 (86.2)	0.0115
3	Is the evaluation of the hereditary-family risk of a tumor carried out as part of the oncological screening pathways?	64 (23.9)	144 (53.7)	108 (40.3)	< 0.0001
4	What is a predictive test?	217 (81.0)	217 (81.0)	209 (78.0)	0.3827
5	What are the main models of Mendelian heritage?	243 (90.7)	252 (94.0)	238 (88.8)	0.4561
6	What do pharmacogenetic tests predict?	210 (78.4)	246 (91.8)	228 (85.1)	0.0290
7	What coverage does participation in cancer screening programs for breast and colorectal cancer reach in our country?	53 (19.8)	93 (34.7)	49 (18.3)	0.6276
8	In hereditary forms of cancer, what is the transmission of the gene involved?	119 (44.4)	146 (54.5)	125 (46.6)	0.5839
9	What types of analysis are performed with Next Generation Sequencing (NGS) techniques?	100 (37.3)	153 (57.1)	144 (53.7)	< 0.0001
10	What is the role of diagnostic genetic tests in the field of hereditary tumors?	204 (76.1)	242 (90.3)	222 (82.8)	0.0366

*The participants who answered T0, T1, and T2 are 268 and the denominator for each question is always 268: in particular, the blank answers were automatically considered as wrong answers, assigning them 0 points.*

**TABLE 6:** Respondents’ answers on the acquired competence to meet the patients request on genetic tests (*N* = 258).

**When asked by patients, I was able to provide information about diagnostic/prognostic utility of predictive genetic test**	Before the course (T0) * N (%)	**After the course (T1) N (%)**	**Eight months after the course closes (T2) N (%)**
Never	22 (8.5)	31 (12.0)	14 (5.4)
Rarely	38 (14.7)	30 (11.6)	22 (8.5)
Occasionally	20 (7.7)	18 (7.0)	16 (6.2)
Sometimes	37 (14.3)	26 (10.1)	21 (8.1)
Regularly	22 (8.5)	23 (8.9)	33 (12.8)
Usually	17 (6.6)	18 (7.0)	20 (7.7)
Always	8 (3.1)	11 (4.3)	23 (8.9)
Not applicable	94 (36.4)	101 (39.1)	109 (42.2)

*Referred to 12 months before the course start.

Regarding knowledge, an improvement was recorded when comparing the correct answers at T1 and at T2 with respect to the pre-test (T0), with the exception of question 4 and 5 (Table 5). The statistical analysis comparing the correct answers given in T2 vs. T0 demonstrated a significant knowledge retain for 6 of the 10 questions (*p* < 0.05) (Table 5).

Table 6 reports the results of the Respondents (*n* = 258 out of 268) to the additional question on the perceived acquired competence about the acquired capability to meet patient requests on genetic tests. According to the answers, their competence improved overall: those who felt less competent were 45.2% at the pre-test, 41% at post-test and 28.2% at follow-up. Respondents who felt more capable of providing information increased from 18.2% in the pre-test to 19.5% and 22.4% in the post-test and follow-up, respectively.

### Satisfaction Questionnaire Results

**FIGURE 2:**
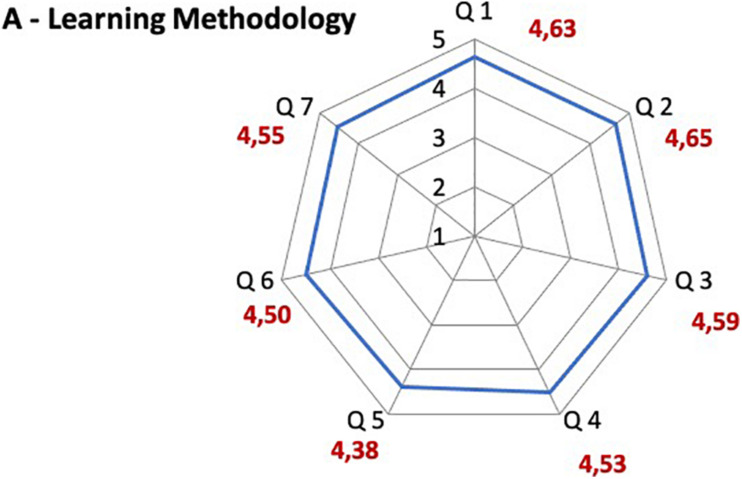
Satisfaction questionnaire results on learning methodology of the course. Q1 The objectives of the course were clear; Q2 The content was consistent with the objectives of the course; Q3 The teaching methodology was effective; Q4 The overall organization (course articulation, timing, intermediate and final evaluations) was satisfactory; Q5 The test questions were clear; Q6 The time available to perform the tests was adequate; Q7 The quality of tutoring for this FAD event was satisfactory.

**FIGURE 3:**
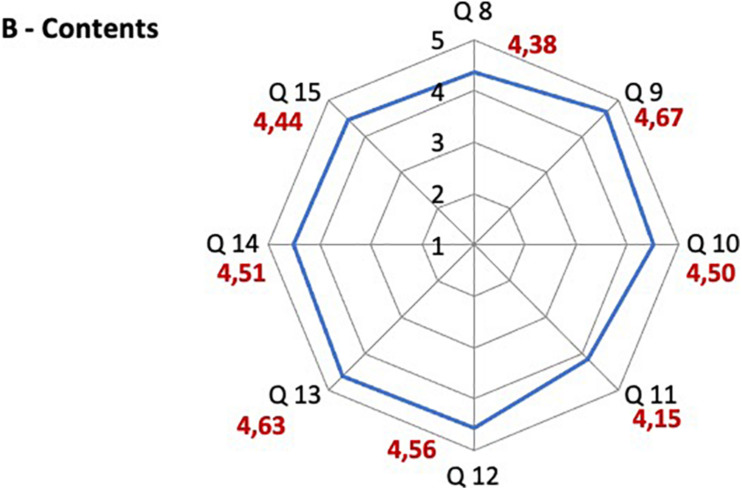
Satisfaction questionnaire results on contents. Q8 The level of the course was appropriate to my knowledge; Q9 I have learned new concepts; Q10 I have acquired new skills; Q11 I can apply what I learned in this course in my working context; Q12 The documentation made available was adequate to acquire the necessary information; Q13 The quality of the documentation available was appropriate; Q14 The documentation available was updated to the most recent literature; Q15 The consultation of the participant’s guide was useful in orienting myself in the educational path.

**FIGURE 4:**
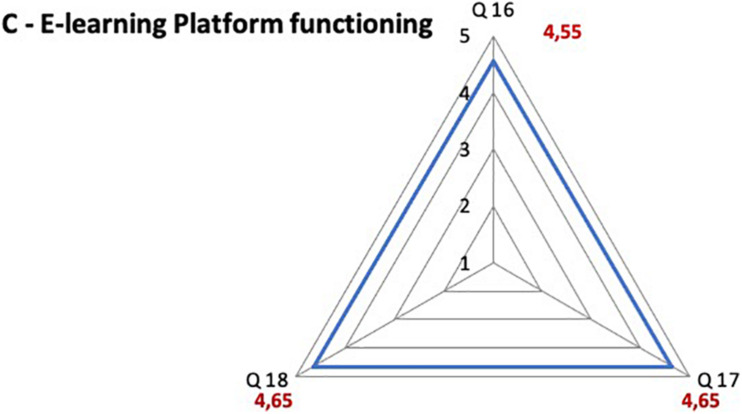
Satisfaction questionnaire results on e-learning platform functioning. Q16 The quality of technical support has been satisfactory; Q17 The functioning of the platform was adequate; Q18 The access to the online platform was simple and immediate.

The majority of participants was satisfied with the learning method, with the adequacy of the contents and with the e-learning platform functioning. A high overall approval on the course emerged, considering that the scores attributed are all between 4 and 5, where 5 expresses the highest degree of satisfaction (Figures 2–4). The question 3 “The teaching methodology was effective”, which aimed to assess the satisfaction on the association of PBL and CBL, scored 4.59 on 5.

## Discussion

The aim of this study was to analyze the effectiveness of a distance learning course on genetics and genomics for Italian physicians. In recent years, a growing interest in promoting courses on genetics/genomics topics has clearly emerged (Kohane, 2015; Cornel, 2019), both due to the rapid developments in genomics technologies and the insufficient knowledge of healthcare practitioners in this field (Feero and Green, 2011; Hall and Luheshi, 2017). A previous research about learning methodologies on genetics suggests that different aspects of an educational intervention may have an impact on its effectiveness, including the type of intervention and the amount of practice-reinforcing strategies it contains (Pastorino et al., 2018). Indeed, interactive learning, including case studies, is generally more effective at improving medical knowledge than learning based on theoretical principles alone (D’Alessandro et al., 2004).

This course represents the second Italian experience in distance training on genomics (Michelazzo et al., 2015).

The main innovative aspect of the “Genetics and Genomics practice” course is related to the teaching methodology, oriented to an active training. The PBL methodology encourages the participants to “learn to learn” by solving real-world problems that reflect their work context (Schmidt et al., 2011; Mazzaccara et al., 2013). Schmidt et al. (Vaona et al., 2018) indicated that in PBL the presentation of a problem activates the participants’ prior knowledge, enabling more effective learning to take place. Compared to a conventional approach, in PBL participants actively attempt to solve a problem and to identify the learning objectives themselves. Therefore, learners face a cognitive conflict and construct their learning on their previous knowledge and experience (Masek and Yamin, 2012). CBL encourages participants to integrate their learning in the context of realistic clinical environment and to connect theory to clinical practice. The learning theories applied to CBL derive mainly from adult-learning and inquiry-based learning approaches, relaying also to cognitive and social constructivist models (Mayo, 2004). Although in some studies CBL is contrasted to PBL in terms of structure, being guided-learning, we integrated these two approaches, in order to provide a comprehensive andragogical and active orientation to the course (Thistlethwaite et al., 2012). Some studies have demonstrated the advantages of either PBL or CBL, but, to our knowledge, there is limited literature that analyzes the association of PBL and CBL methods, especially with respect to genomics topics and to the e-learning context (Zhao et al., 2020). Assuming that the two methods are both effective and suitable in medical education and that CBL better accomplishes the course’s clinical orientation, we associated the PBL and CBL teaching methods in order to complement and reinforce each other. The high score of the SQ’s question “The teaching methodology was effective” supports our choice to associate PBL with CBL. Nevertheless, the evaluation of the effectiveness of the PBL/CBL model would require an analysis between courses based only on PBL or CBL compared to those based on the combined model. We have planned to develop such an analysis in the future courses.

The results of our “Genetics and Genomics practice” course suggest that distance-learning training in genetic/genomics practice represents an effective and satisfactory method to improve physicians’ knowledge across all age groups of participants. Among the 1,637 participants who completed the course, the most represented age class was those of 51–65 years. This may be related to the educational need of the over-50 age physicians in an innovative field as omics sciences. In fact, most healthcare professionals had not received adequate training on this topic during their studies, as demonstrated by the negative correlation between time from medicine degree and omics sciences knowledge (Hofman et al., 1993). In our sample of participants, the majority (79.4%) declared it was their first course on genetics/genomics, thus making us quite confident about limiting a possible bias on results due to previous training on these topics.

The effectiveness of the course was measured through a test made of a set of 10 MCQs that was repeated before the start and at the end of the course. The overall results suggest that the course improved the general level of knowledge. Nevertheless, as revealed by the stratified analysis, the improvement was not homogeneous for all the medical specialties. For example, the knowledge improvement was greater for Primary Care physicians and Sports Medicine physicians. These results accomplish our expectations regarding the course, since it was intended mainly as directed to GPs and FPs (primary care), that usually don’t receive a specific education in genetics during their specialization, but deal with genetic disorders during their daily practice. The low pre-test score of this specialty group confirmed the educational need we hypothesized in planning the course. As for Sports Medicine physicians, the lowest pre-test score they reported could be explained with the fact that they deal with genetics more rarely than other specialists; however, the great improvement obtained in the post-test score could demonstrate the effectiveness of the course in filling the knowledge gap. On the opposite, the Gynecologists and Occupational Medicine physicians reported the lowest difference between the pre and post-test. For the first category, this might be related to the high pre-test score they reported, while for the second both the pre-test and the post-test scores were low, if compared to the overall scores. The highest pre-test and post-test scores were registered for the Geneticists, demonstrating their pre-existing knowledge on the topic. Our results are consistent with those reported in the study of Michelazzo et al. that had analyzed the effect of a course in genetics and genomics for physicians, organized with different educational methodologies (Michelazzo et al., 2015).

To our knowledge, this is the first study that attempts to measure the knowledge retain and the educational effects of a genetics/genomics after a follow-up period. This was obtained by inviting all participants to complete the same pre/post-test after 8 months. Although the number of Respondents was low if compared to the high number of the course completers (268 vs. 1,637), we can assume that this group is representative of the Completers, as the demographic and professional characteristics of the two groups were analogous.

The overall scores show that after 8 months the knowledge level decreased if compared to the post-test score, but it was higher than the pre-test score. The statistical analysis comparing T2 vs. T0 demonstrated an overall significant increase and retain in knowledge.

The follow-up data also allow some considerations on the self-perceived sense of competence of the medical professionals in giving information on genetic tests to patients, before and after the course. According to our results, the sense of competence improved at follow-up and there was an increase in the number of doctors who felt more capable of providing information about the diagnostic/prognostic utility of genetic tests.

Our study presents some limitations. Firstly, the effectiveness of the course could be overestimated, since only data of those who completed the entire course were collected. Therefore, it might be possible that the “dropouts” would have reported lower improvements or less satisfaction than the “completers.” Secondly, the sample size of those who completed the course was quite heterogeneous in terms of specialties, not allowing a significant representation of all the discipline categories, many of which were grouped into one “other specialties” category.

Despite these limitations, the results of our study confirmed the effectiveness of genetic and genomics courses in improving participants’ literacy on omics sciences, not only in terms of knowledge, but also in terms of managing genetic information in daily practice. In particular, our results suggest that especially primary care physicians are those who can have the most important benefit from a course on this topic.

The course was characterized by some innovative aspects. First of all, as strongly requested by the Italian Ministry of Health, the course was direct to all the medical specialties, despite the specificity of such a current topic. Indeed, for the first time, a large number of various health professionals could access a learning project on genetics and genomics, not reserved to specialists in genetics only. Moreover, we experimented a new educational approach, consisting of a synergy between two different approaches, PBL and CBL.

## Conclusion

In conclusion, in our experience, a distance-learning training in genetic/genomics practice that adopted a PBL and CBL approach was highly effective in improving physicians’ knowledge and self-perceived competence on those topics.

## Data Availability Statement

The raw data supporting the conclusions of this article will be made available by the authors, without undue reservation.

## Author Contributions

SB, WR and GEC conceived the study. GEC, AT, AM, and AF participated in its design. SB, GEC, and AM developed the content of the distance learning course. AM, PC, DB, and ADP realized the course in the online platform and collected the participants’ data. AT, GEC, AM, PC, DB, and ADP statistically analyzed the data. SB, GEC, AT, AM, PC, DB, and ADP critically discussed and interpreted the results of the knowledge tests. All authors drafted and critically reviewed this manuscript and approved the final version.

## Conflict of Interest

The authors declare that the research was conducted in the absence of any commercial or financial relationships that could be construed as a potential conflict of interest.

## Footnotes

1 https://www.eduiss.it
